# The greatest happiness of the greatest number? Policy actors' perspectives on the limits of economic evaluation as a tool for informing health care coverage decisions in Thailand

**DOI:** 10.1186/1472-6963-8-197

**Published:** 2008-09-26

**Authors:** Yot Teerawattananon, Steve Russell

**Affiliations:** 1 Health Intervention and Technology Assessment Program (HITAP), Ministry of Public Health, Nonthaburi, Thailand; 2 School of Development Studies, University of East Anglia, Norwich, UK

## Abstract

**Background:**

This paper presents qualitative findings from an assessment of the acceptability of using economic evaluation among policy actors in Thailand. Using cost-utility data from two economic analyses a hypothetical case scenario was created in which policy actors had to choose between two competing interventions to include in a public health benefit package. The two competing interventions, laparoscopic cholecystectomy (LC) for gallbladder disease versus renal dialysis for chronic renal disease, were selected because they highlighted conflicting criteria influencing the allocation of healthcare resources.

**Methods:**

Semi-structured interviews were conducted with 36 policy actors who play a major role in resource allocation decisions within the Thai healthcare system. These included 14 policy makers at the national level, five hospital directors, ten health professionals and seven academics.

**Results:**

Twenty six out of 36 (72%) respondents were not convinced by the presentation of economic evaluation findings and chose not to support the inclusion of a proven cost-effective intervention (LC) in the benefit package due to ethical, institutional and political considerations. There were only six respondents, including three policy makers at national level, one hospital director, one health professional and one academic, (6/36, 17%) whose decisions were influenced by economic evaluation evidence.

**Conclusion:**

This paper illustrates limitations of using economic evaluation information in decision making priorities of health care, perceived by different policy actors. It demonstrates that the concept of maximising health utility fails to recognise other important societal values in making health resource allocation decisions.

## Background

In all societies health care resources are restricted so that priority setting for health expenditure has to be done either implicitly or explicitly[1]. Health economic evaluation is a method used to analyse the costs and benefits of different health care interventions, and has often been quoted as the most promising tool to assist decision-makers in health care rationing[2,3]. Cost-utility analysis, which is one type of health economic evaluation, is widely recommended in many official health technology assessment guidelines in many settings [4-7]. The method assumes that the ultimate goal of the health care system is to maximise health benefits from the finite resources available, regardless of the distribution of these health benefits. To allow comparisons across a broad spectrum of intervention or programme areas, a common health benefit composite indicator, such as the Quality Adjusted Life Year (QALY), has been created and applied to numerous interventions to enable decision makers to decide which health investments maximise health (QALYs)[8,9]. A QALY measures both the quantity of life generated by an intervention (in years) and the change to quality of life in each of those years.

Although there are several moral and methodological controversies over the use of economic evaluation to guide health resource allocation[3,10,11], it is increasingly being used in some industrial countries on the grounds that it can inform more explicit and transparent health care coverage decisions[12]. In low- and middle-income countries the tool has rarely been used to inform decisions about the content of health care benefit packages. However in middle income countries such as Thailand policy-makers are facing growing pressure to justify resource allocation decisions in the health sector, due to increasing resource constraints arising from an epidemiological transition and increases in the availability and cost of new health technologies [13-15]. In Thailand the Universal Health Insurance Coverage (UC) policy implemented in 2001 offers a package of health care interventions at public facilities to all Thai citizens not covered by other benefit packages[16]. There is growing pressure on the government to clarify and make more transparent the UC benefit package, particularly for high cost interventions that absorb a disproportionate amount of resources[17]. Some high cost interventions are included in the package, others are excluded and some are in a 'grey zone' and provided at the discretion of consultants or hospital directors. A mix of criteria, mainly implicit, have influenced these decisions, for example pre-existing service availability, affordability for the provider and political pressures[18].

This paper presents qualitative findings based on semi-structured face-to-face interviews that explored the acceptability of using only evidence from economic evaluation among different policy actors. A case scenario was constructed using information from two separate economic evaluation studies previously conducted in Thailand. One was an economic evaluation of open versus laparoscopic cholecystectomy for gallbladder stone disease[19] and the other was an economic evaluation of renal dialysis compared to palliative treatment of end-stage renal disease[20]. The interviews sought to explore policy actors' justifications for their decisions on the case scenarios, including the trade-offs they had to make between cost utility criteria founded on the principle of health (QALY) maximisation, and other criteria such as disease severity and overall budget impact[21,22].

## Methods

### Respondents

The selection of respondents was purposive to cover four groups of policy actors who play a major role or influence in health resource allocation decisions within the Thai healthcare system. A purposive sampling strategy was used to ensure that a range of policy actors was covered and that, at the highest level, the most important policy actors were selected. The qualitative data generated is not intended to be 'representative' in statistical terms, but the data can be used to build understanding of policy actors' attitudes and positions relating to economic evaluation in decision-making. Depth of understanding rather than sample size was the main concern[23,24]. However the policy relevance of the findings did rely on ensuring that an appropriate range of policy actors for this particular setting were covered, to enable the capture of a 'typical' range of perspectives[25].

As a result, an invitation letter, research proposal and consent form were sent to each of 38 potential participants including:

• fourteen **policy makers at the national level **who were the most senior administrators at the Ministry of Public Health (MOPH) and National Health Security Office (NHSO), which is an autonomous health care institution in Thailand that manages the Universal Health Coverage scheme;

• five **hospital directors **who are responsible for allocating resources within Thai healthcare institutions;

• twelve **health professionals **(medical specialists) who are responsible for resource allocation decisions at the patient-level, and;

• seven **academics **who produce and/or use economic evaluation information to inform decision makers.

For policy makers at national level letters were sent to the top seven senior administrators at the MOPH, both politicians and bureaucrats, and the top seven senior administrators of the NHSO. For the hospital directors, the five directors of the public hospitals where the authors had previously conducted the aforementioned economic evaluation studies[19,20] were invited for interview. The invitation letters were also purposively sent to health professionals at those five public hospitals. It was an intention to cover a wide range of medical specialists including two internists, two surgeons, two nephrologists, two paediatricians, one oncologist, one ophthalmologist, one orthopaedist and one otorhinolaryngologist. Finally, seven Thai academics whose names were identified from national and international publications on issues of 'health care rationing/prioritisation' were invited to participate in the study.

Thirty-six respondents agreed to participate and were interviewed between December 2004 and May 2005 (missing two health professionals, paediatrician and orthopaedist). They were predominantly male (only two were female physicians), had an average age of 50 years and 34 out of 36 (94%) had a medical background (only two academics not qualified in medicines), which reflects the composition of senior management in the health sector in Thailand more generally. Only two policy makers and four academics had formal training in health economics or health care financing.

### Interview schedule

At the beginning of the interview every respondent was given a brief introduction to health economic evaluation, including the concepts and applications of QALY maximisation. The semi-structured interview schedule then had two related parts. The first was a set of questions to explore policy actors' opinions about existing criteria for including health interventions in the UC benefit package, and their acceptance and values relating to the use of economic evaluation for development of the benefit package. The findings from these general questions are presented elsewhere[26].

The second part of the interview consisted of a hypothetical decision-making case scenario in which respondents were presented with a choice of two interventions and asked to decide which one to include in the UC package, based on different types of evidence, including the economic evaluation data collected as part of the wider research project. They were given a scenario in which the government was considering inclusion of one of two treatments, (1) laparoscopic cholecystectomy (LC) for gallbladder disease, versus (2) dialysis for chronic renal disease. The data presented to the respondents came from the results of economic evaluation studies conducted by the first author[19,20].

The selection of the two interventions for the case scenario was based on several important factors. Firstly, it was important to make the hypothetical scenario as realistic as possible, and both these treatments were the subject of real public debate at the time of the study. There was and continues to be pressure from various interest groups to include dialysis for chronic renal disease and laparoscopic surgery in the UC benefit package[27]. Neither LC nor dialysis were covered by the UC at the time of the interview, although conventional open cholecystectomy (OC) for gallbladder disease and palliative management for chronic renal disease were included. LC and dialysis were both being offered by other public health insurance schemes at the time.

Secondly, the two interventions were selected because they have several features, identified from the literature, which were likely to highlight conflicting priorities towards the allocation of health care resources (see Table 1), and so stimulate discussion about the application of economic evaluation in real world decision-making, for example whether life saving interventions should be prioritised over cost effective interventions, and how to deal with questions of equitable resource allocation or protection against catastrophic health care payments[28].

**Table 1:** Comparison of characteristics of laparoscopic cholecystectomy (LC) and renal dialysis used in the case scenario.

	**Severity of disease and importance of the intervention: are there alternatives?**	**Equity of access improvement**	Cost-effectiveness based on economic evaluation*	**Financial impact on government budget**
	+	++	+++	-
**LC for gallbladder disease**	Medical treatment and open conventional (OC) surgery are both available.	13% of patients in the country undergoing LC are under UC but have to pay a proportion of the cost. An alternative (OC) is available without a charge.	Compared to open surgery, the incremental cost-effectiveness ratio (ICER) for LC is less than 1 Thai GDP per capita and so considered cost-effective.	Relatively very small budget needed if it is to be included in the UC package. If included the indirect and direct non medical costs to households would also be reduced substantially.

	+++	+++	-	+++
**Dialysis for end-stage renal disease**	The availability of kidney donors is very limited. Without dialysis or kidney transplantation patients will die within 3–6 months.	Less than 5% of patients undergoing dialysis are under UC and have to pay the full cost. There is no alternative available for them.	Compared to 'palliative care', ICER for dialysis is higher than 5 times Thai GDP per capita and so considered non cost-effective.	Very huge financial impact on the overall UC budget.

*A report from the Commission on Macroeconomics and Health suggests the use of a threshold three times that of Gross Domestic Product (GDP) per capita as a basis for interpreting whether an intervention is cost-effective and should be adopted as a health technology in developing countries [32].

Marks: +++ "very high", ++ "high", +"moderate", – "none".

In order to assess the relative importance given by respondents to a particular type of information or evidence (disease severity and treatment alternatives, cost effectiveness, budget impacts) the information was deliberately not presented at once but arranged into three staged components. Each piece of information was revealed separately and between each presentation the respondent was asked to choose the intervention that they would support to be included in the UC package. In addition, the interviewer did not inform respondents that there would be more information available after presenting the first and then the second piece of information.

The first piece of information described the two treatments and the expected recovery rates or quality of life resulting from the treatment [see additional file 1]. The second piece of information described the cost utility ratios of the two interventions, to see if this information changed the respondent's decision to choose between LC for gallbladder disease or dialysis for chronic renal disease [see additional file 2]. Finally, the overall financial impacts for the government and patients were presented [see additional file 3]. It was expected that the financial implications for both public and private sectors would have a greater influence on the respondent's decision than economic evaluation information, so these financial implications were presented last.

After each piece of information was presented, a structured question was asked to elicit a specific decision-making response. To encourage a response to the case scenario the interviewer stressed that there were no right or wrong answers. Although the respondents could refuse to make a choice, this option was not openly expressed to them so the refusal to make a choice was accepted only on request. Following the structured choice question, respondents were then encouraged to discuss and explain their decisions using open question formats.

### Analysis

All interviews were recorded on audiotape and transcribed verbatim. The first author read all the Thai transcripts and developed a list of codes (or themes) and sub-codes that were derived from respondents' understanding and reasoning behind their choices. One of our interests was to explore whether the respondents' different positions and duties influenced their attitudes and acceptance of using economic evaluation as a tool for healthcare rationing. The analysis also consisted of simple descriptive statistics (absolute counts and percentages) to describe policy actors' choices.

## Results

**Figure 1:**
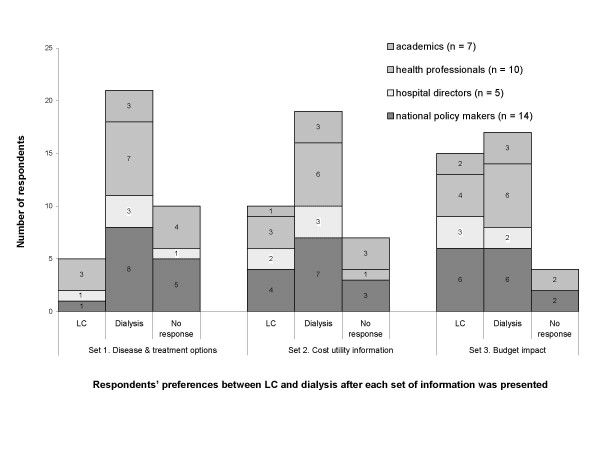
Distribution of choices by type of respondent after three sets of case scenario information were presented sequentially.

The distribution of responses to the three pieces of information is shown in [see Figure 1]. Given the first information set about disease severity and treatment, 58% of respondents, including eight decision makers at national level, three hospital directors, seven health professionals and three academics opted to support the life-saving intervention, dialysis for chronic renal disease, rather than LC for gallbladder disease. The most common explanation from the supporters was that dialysis was a life-saving intervention, whereas LC was not life saving and without LC conventional open surgery was still effective and available to patients.

One academic respondent supported dialysis on the grounds that including it in the UC benefit package would reduce an inequality of access in the Thai health care system:

"I preferred dialysis because at present all health insurance schemes except the UC provide dialysis to their beneficiaries" (academic).

A small group (one decision maker at national level, one hospital director and three health professionals) chose to reject dialysis and support LC, but mainly for reasons other than cost-effectiveness. The one policy maker believed that dialysis would not be a cost-effective option while the hospital director and the three health professionals believed that there would not be adequate financial or human resources, for example nephrologists and dialysis nurses to provide adequate dialysis, if the UC included it within the benefit package:

"The government can spend money to buy dialysis machines right now as much as they want but they can't buy hundreds of nephrologists and nurses over night" (hospital director).

Five decision makers at the national level, one hospital director and four academics refused to make a choice at this stage and asked for more information on cost-effectiveness.

When the second information set was provided (economic evaluation findings), five respondents shifted to the LC (Figure 1). One policy maker at the national level shifted support from dialysis to LC and explained:

"If I was looking at an individual patient I would choose dialysis, but I am currently making this decision for society as a whole and evidence shows that LC is a better choice" (national policy maker).

The other four who shifted their support to LC came from the group of ten who had previously not made a decision (two policy makers, one hospital director and one academic). They argued that the economic evaluation data were good enough to justify support of LC:

"If these figures are right, it's clear that dialysis is cost-ineffective so I would not support it" (academic).

One policy actor, a health professional, moved from supporting dialysis to a no response after hearing the cost-effectiveness information and explained that her decision was based on confusion:

"I know it [dialysis] is very important for patients with renal disease but your data make me feel that it may be too expensive to extend their life. I am now confused and not sure whether to support either" (health professional).

Despite the cost effectiveness information being presented, however, about half of the respondents (19 or 53%) continued to support dialysis. Most of them felt that in this particular situation it was ethically wrong not to support dialysis that could save a number of lives:

"In my view, your choices (LC versus dialysis) are incomparable.... Even if the treatment proves to be cost-ineffective, not covering it might create the impression that critically ill patients are being abandoned" (national policy maker, senior administrator of NHSO).

Three decision makers at the national level also thought that the coverage decision should be made not only on theoretical and empirical grounds but importantly it should also make sense and be acceptable to the general public. Since any decision made by them would be announced to the public they argued that it should be politically defensible. In this case, they thought that it was unacceptable to let patients with chronic renal disease die without offering proven effective treatment. They felt that because dialysis was a life-saving intervention, the general public would opt to support it over LC and so they would also like to do so:

"If the UC announces to the public that it will include LC in the benefit package, I think that it will not be appreciated by many people. But if the UC is about to cover dialysis, it will be very much different" (national policy maker).

The decision shift away from dialysis to LC was most dramatic after the third information set was presented: the global budget impact of their decision. Three respondents shifted from a no response situation to LC (one policy maker at national level, one academic and one health professional), and two shifted from dialysis to LC (one policy maker at national level and one hospital director). Both of the latter explained they were now aware that the total cost of their decision to support dialysis was too expensive for the Thai healthcare system and that the government budget was too limited for dialysis in the long run.

After all three pieces of information had been presented more respondents (n = 17) still supported dialysis over LC (n = 15), despite LC's higher cost effectiveness. Four respondents still refused to make a choice for several reasons: both choices were not attractive and some alternative options were needed; the public should play a part in making this tough decision; and there was still not enough information to make a decision, for example the lack of cost-utility information for a range of potential interventions that needed to be considered at the same time:

"We can't consider only two interventions. Theoretically, we need to compare altogether all interventions that are in and out of the package since we may find some interventions outside the package that are more cost-effective than these two" (academic).

## Discussion

Cost-utility analysis is well accepted amongst health economists, given the number of publications in academic journals[29], but little is known about how policy makers and health professionals perceive and value its findings and whether such evidence is meaningful to them and relevant to the decisions they take[30]. The present study qualitatively illustrated how different health policy actors react to decision-making dilemmas about resource allocation, for example whether to give priority to cost-effective non-life saving interventions, or cost-ineffective life saving interventions.

The data presented on policy actors' responses when faced with a hypothetical but realistic decision confirms that health care policy actors saw limits to the usefulness of economic evaluation (cost-utility analysis)[31]. Twenty six out of 36 (72%) respondents were not convinced by the presentation of economic evaluation findings and chose not to support the inclusion of a proven cost-effective intervention in the UC benefit package. Even the majority of academics supported renal dialysis due to ethical or equity concerns. Indeed, there were only six respondents, including three policy makers at national level, one hospital director, one health professional and one academic, (6/36, 17%) whose decisions were influenced by economic evaluation evidence.

It seems reasonable to conclude that economic evaluation does not deal with many important factors or priorities that concern decision makers when they are making rationing decisions:

• ethical concerns relating to questions of saving life or equity;

• the availability and accessibility of treatment alternatives;

• awareness of the feasibility of policy options including availability of human and financial resources;

• organizational allegiances and institutionalised practices such as the primacy of the right to treatment;

• concerns about power over decision-making and wider political pressure on policy makers[31].

The findings presented in this paper add substance to and illuminate these complexities and difficulties. One of the most obvious difficulties is that economic evaluation ignores alternative ethical values that can be held by policy actors. More respondents, for example, decided that it was ethically right to prioritise a life-saving cost-ineffective intervention, dialysis, over a non-life saving cost-effective intervention, LC. This ethical preference clearly conflicts with economic evaluation, which is founded on a health maximisation philosophy, and echoes well-founded ethical positions that point to the importance of helping the neediest as the basis of philosophical justice[28,32,33]. Policy actors who prioritised severely ill candidates ahead of others, even though their treatment was less cost-effective, also argued that the majority of the public would have the same ethical values and expectations for healthcare rationing. In other settings studies have explored public preferences towards the use of the QALY maximisation rule, and found that the general public's view does not always support maximising the number of QALYs gained[33]. People were willing to prioritise resource allocation to severely ill patients, even when they would benefit less from treatment than others, or were willing to reduce the number of QALYs gained in order to help those perceived to be most in need in terms of severity of illness.

Policy makers' concern about the public's support for QALY maximisation highlights the political factors that influenced coverage decisions in the case scenario. Among decision makers at the national level, despite their expressed concern about resource constraints and the need for efficiency, not all supported the cost-effective LC intervention because they were aware of the importance of gaining public support and acceptance of their decision. Their career paths are, to some extent, dependent on their ability to justify and defend their decisions politically and gain public acceptance.

Hospital directors, in contrast, had fewer concerns about public perceptions and by the end of the interview the majority (3/5, 60%) had rejected dialysis and supported LC. However the support for LC from two of these three directors was based on overall resource constraints rather than on the health maximising concept of economic evaluation, reflecting their responsibility for the management of the hospital's financial and human resources to deliver services.

Health professionals' are trained and operate within an institutional environment that means in principle they act in the best interest of the patient, so they are likely to prioritise patient care over economic considerations. This helps to explain why the majority (6/10) continued to support dialysis after the presentation of economic evaluation information. The majority of health professionals were more concerned about saving lives, even when the opportunity cost was a reduction in the quality of life of other individuals in need. This decision perhaps reflects the fact that health professionals' overriding professional responsibility is to the particular patient under consideration[34], and that they make decisions for individuals with less recourse to wider societal perspectives than the national policy makers.

Even in the case of academics trained in economic evaluation, more did not support the use of economic evaluation for prioritising healthcare than did. While they argued that improved efficiency through the use of economic evaluation was important they also stressed that this criterion needed to be balanced against equity and affordability. This illustrates the fact that the non-use or selective use of economic evaluation will not simply be resolved by providing appropriate education of information but incorporate various competing decision making priorities in order to gain widespread acceptance in the priority setting process.

It is important to note two possible limitations of these findings. First, the data on policy actors' decisions are based on a hypothetical scenario and in a real world scenario the decisions made may well have been different. For example in Thailand decision makers might look at just one intervention such as dialysis and consider affordability and cost-effectiveness, but not make comparisons across health problems. However, the scenario presented was a topical and realistic one. All the information provided, including the economic evaluation data and financial implications, were based on real studies and the case of dialysis was one of public debate at the time of the interviews because the government was considering its inclusion in the UC benefit package[35]. During the interviews it was evident that the respondents took the questions very seriously. Hence the decisions made in the hypothetical situation may, in fact, reflect the real preferences of respondents if they had been taking part in a real policy decision.

Second, this study was not undertaken to produce 'generalisable results' about how economic evaluation might be accepted or used in other settings. Decision makers elsewhere may attach more or less weight to different resource allocation criteria, and the same health technology may have different characteristics where it is offered in other settings. Also, it is not possible to generalise the findings from this study to all policy makers in Thailand. However, the qualitative design aimed to offer in-depth understanding about the complexity of decision-making in a specific context which can still be informative for analysts elsewhere.

## Conclusion

The policy actors' perspectives and positions, presented in this and a related paper[31] have highlighted several difficulties and dilemmas for the introduction of economic evaluation into health technology coverage decision making processes in Thailand. There was a lack of consensus between and within different groups of health care policy actors on the best criteria for allocating scarce health care resources. However, interpreting the data on policy actors' different priorities and decisions, and the rationales behind them, it is possible to better understand the different priorities of policy actors and so inform better procedures for or management of a complex and unavoidable rationing process in healthcare.

Increasing the use of economic evaluation in Thailand, to make health technology resource allocation decisions more explicit and transparent, requires a search for how best to incorporate the tool within existing and competing decision making priorities. Otherwise, economic evaluation which is based mainly on a concept of 'the greatest happiness of the greatest number' would fail to provide a guide for making rational resource allocation in most cases.

## Competing interests

The authors declare that they have no competing interests.

## Authors' contributions

YT designed the study, carried out the data collection and analysis, and drafted the paper. SR participated in the study design, advised on the collection of data and made substantial contributions to the data interpretation and writing of the paper. All authors read and approved the final manuscript.

## Pre-publication history

The pre-publication history for this paper can be accessed here:



## Supplementary Material

Additional file 1 **The first set of information: the two treatments and the expected recovery rates or quality of life.** Click here for file

Additional file 2 **The second set of information: the cost utility ratios of the two interventions.** Click here for file

Additional file 3 **The third set of information: overall financial impacts for the government and patients.** Click here for file
