# Supplementary material for: The greatest happiness of the greatest number? Policy actors' perspectives on the limits of economic evaluation as a tool for informing health care coverage decisions in Thailand
Source: BMC Health Serv Res. 2008 Sep 26;8:197. doi: 10.1186/1472-6963-8-197 (PMC2569929; doi:10.1186/1472-6963-8-197)
Supplement: Additional file 1 — The first set of information: the two treatments and the expected recovery rates or quality of life. [file 1472-6963-8-197-S1.doc]

The first set of information: the two treatments and the expected recovery rates or quality of life.

Laparoscopic cholecystectomy(LC) is a minimal invasive treatment for gallbladder stone disease. Compared to the conventional open cholecystectomy(OC), LC is associated with a 3 or 4 day-shorter hospitalization, 2-3 week more rapid return to work and 10% better quality of life one year after the operation.

Hemo- and peritoneal-dialyses are treatments for people with chronic kidney disease (CKD). Without treatment those with CKD will commonly die within 3 months but with dialysis 20 year-old patients with CKD would live 18 more years or 65 year-old patients would live 7 more years. The nature of dialysis treatment means that it is required regularly either at home every day in the case of peritoneal dialysis, or at the hospital or a center for those receiving hemodialysis 2 or 3 times a week. The quality of life of those with dialysis is around 70% of that of healthy people.

Currently, LC and hemodialysis are not included in the UC benefit package but OC and palliative treatment for CKD are. In Thailand each year there are around 10,000 people needing LC and 10,000 people needing dialysis.

Which treatment would you prefer to prioritise? And what are the reasons supporting your answer?
