# Supplementary material for: The greatest happiness of the greatest number? Policy actors' perspectives on the limits of economic evaluation as a tool for informing health care coverage decisions in Thailand
Source: BMC Health Serv Res. 2008 Sep 26;8:197. doi: 10.1186/1472-6963-8-197 (PMC2569929; doi:10.1186/1472-6963-8-197)
Supplement: Additional file 2 — The second set of information: the cost utility ratios of the two interventions. [file 1472-6963-8-197-S2.doc]

The second set of information: the cost utility ratios of the two interventions.

You now know that the incremental cost per quality adjusted life year of providing LC (replacing OC by LC) is 86,000 Baht and the incremental cost per quality adjusted life year of providing dialysis (replacing palliative treatment by either of dialysis modality) is around 700,000 Baht.

Which treatment would you prefer? And what are the reasons supporting your answer?

Note: The case study put aside certain technical questions such as how costs and benefits were counted by assuming that all information was obtained currently and correctly. Only simple incremental cost-effectiveness ratios, without expressing a degree of uncertainty, were given to respondents in a scenario. This does not mean that we ignored methodological standards for the evaluations, but only for simplicity and time-constraints during the interviews.
