# Supplementary material for: The greatest happiness of the greatest number? Policy actors' perspectives on the limits of economic evaluation as a tool for informing health care coverage decisions in Thailand
Source: BMC Health Serv Res. 2008 Sep 26;8:197. doi: 10.1186/1472-6963-8-197 (PMC2569929; doi:10.1186/1472-6963-8-197)
Supplement: Additional file 3 — The third set of information: overall financial impacts for the government and patients. [file 1472-6963-8-197-S3.doc]

The third set of information: overall financial impacts for the government and patients.

Implementing LC would cost 77 million Baht to the government but save 31 million Baht to the households affected, so that the net societal cost is 46 million Baht each year.

On the other hand providing dialysis for all patients with CKD would need an additional 6,000 million for the first year, 28,000 million for the fifth year, and 74,000 million for the twentieth year after implementation.

Which treatment would you prefer? And what are the reasons supporting your answer?
